# Supplementary material for: Epigenetic Mechanisms of Postoperative Cognitive Impairment Induced by Anesthesia and Neuroinflammation
Source: Cells. 2022 Sep 21;11(19):2954. doi: 10.3390/cells11192954 (PMC9563723; doi:10.3390/cells11192954)
Supplement: Supplementary file 1 [file cells-11-02954-s001.zip › cells-1898044-supplementary.pdf]

cells

MDPI

Review

Epigenetic Mechanisms of Postoperative Cognitive Impairment Induced by Anesthesia and Neuroinflammation

Katharina Rump * and Michael Adamzik

Department of Anesthesiology, Intensive Care Medicine and Pain Therapy, University Hospital Knappschaftskrankenhaus Bochum, Ruhr-University Bochum, In der Schornau 23-25, 44892 Bochum, Germany

* Correspondence: katharina.k.rump@rub.de; Tel.: +49 234-3229242

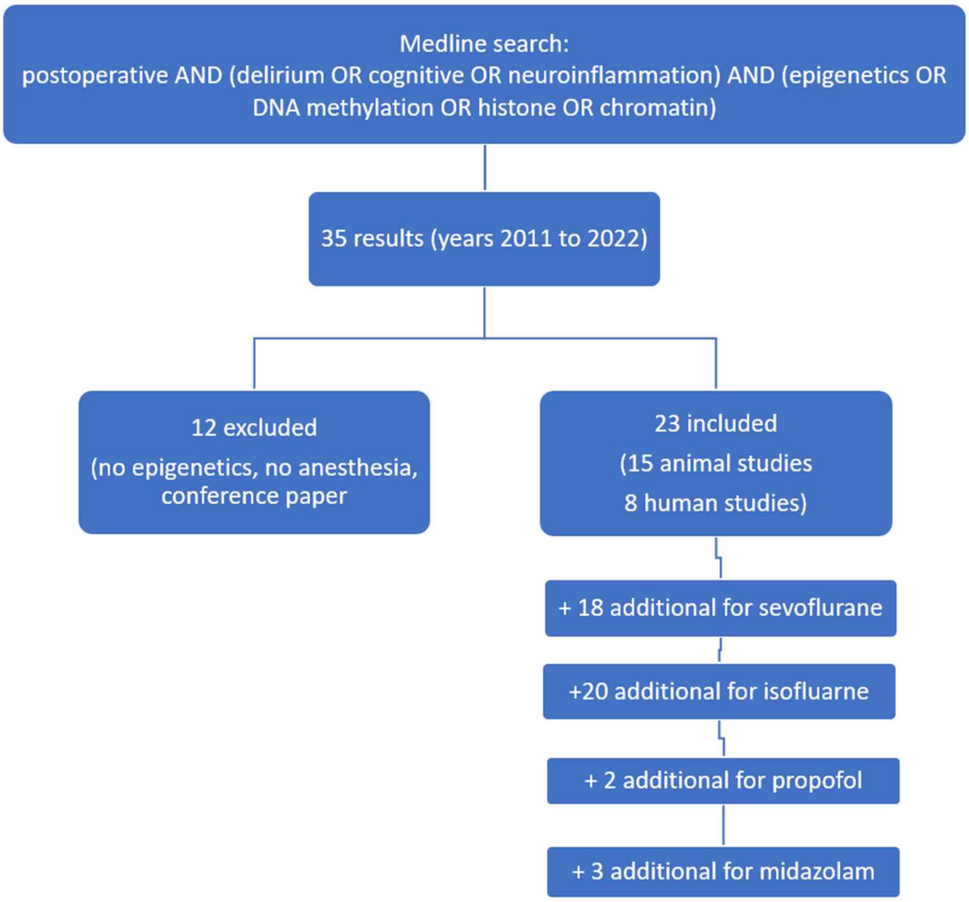

Figure S1. Schematic overview of workflow of literature research: We searched medline for the following search terms: "postoperative AND (delirium OR cognitive OR neuroinflammation) AND (epigenetics OR DNA methylation OR histone OR chromatin)" and search term for specific anaethetics: "(midazolam OR propofol OR isoflurane OR sevoflurane OR postoperative) AND (delirium OR cognitive OR neuroinflammation) AND (epigenetics OR DNA methylation OR histone OR chromatin)". This search strategy let to 78 results in total, whereof 12 were excluded.
